# Interdisciplinary Research in Alzheimer’s Disease and the Roles International Societies Can Play

**DOI:** 10.14336/AD.2020.0602

**Published:** 2021-02-01

**Authors:** Shawn Zheng Kai Tan, Robert Chunhua Zhao, Sasanka Chakrabarti, Ilia Stambler, Kunlin Jin, Lee Wei Lim

**Affiliations:** ^1^Neuromodulation Laboratory, School of Biomedical Sciences, Li Ka Shing Faculty of Medicine, The University of Hong Kong, Hong Kong SAR, China.; ^2^International Society on Aging and Disease (ISOAD), Fort Worth, Texas, USA.; ^3^The Executive Committee on Anti-aging and Disease Prevention in the framework of Science and Technology, Pharmacology and Medicine Themes under an Interactive Atlas along the Silk Roads, UNESCO, Paris, France.; ^4^School of Life Sciences, Shanghai University, Shanghai, China.; ^5^Department of Biochemistry and Central Research Cell, M M Institute of Medical Sciences and Research, Mullana, India.; ^6^The Geriatric Medical Center "Shmuel Harofe", Beer Yaakov, affiliated to Sackler School of Medicine, Tel-Aviv University, Tel-Aviv, Israel.; ^7^Department of Pharmacology & Neuroscience, University of North Texas Health Science Center, Texas, USA.

**Keywords:** Alzheimer’s disease, international societies, interdisciplinary, research

## Abstract

An ever-increasing ageing population has elevated Alzheimer’s disease to be one of the biggest challenges in modern medicine. Alzheimer’s disease is highly complex, and we are still no closer to understanding the causes, let alone an effective treatment. The lack of good experimental models and lack of critical understanding has led to high failure rates of clinical trials with high associated costs, as well as difficulties in implementing treatments. The multifaceted nature of this disease highlights the need for an interdisciplinary approach to address these concerns. In this essay, we suggest how collaborative work can be useful in addressing some of the above issues. We then propose that international organisations and publishers need to support interdisciplinary research by creating platforms, lobbying funders, and pushing for interdisciplinary publications. We further highlight some of the issues involved in implementing these suggestions and argue that willpower of the research community, together with a re-evaluation of evaluation metrics and incentive systems, are needed in order to foster interdisciplinary research. Overall, we emphasise the need for interdisciplinary research in Alzheimer’s disease and suggest that international societies should play a huge role in this endeavour.

The increasingly ageing population poses major challenges worldwide, particularly aging issues such as disease and healthcare. Dementia affects an estimated 35.6 million people worldwide, and Alzheimer’s Disease (AD) accounts for up to 75% of all diagnosed dementia cases [1]. The number of people with dementia is expected to more than triple by 2050, which will inevitably have enormous social and economic costs [2]. The experimental research in AD has been highly skewed in favor of Amyloid Cascade Hypothesis and the use of transgenic AD models [3, 4]. At the same time a huge body of epidemiological data on various metabolic and environmental risk factors of AD has been somewhat ignored to develop suitable models or to formulate new treatment strategies for AD [5]. Thus, identification of various associated risk factors of AD such as type 2 diabetes, hyperhomocysteinemia, hypercholesterolemia, hypovitaminosis D, altered levels of pro-inflammatory cytokines, adiponectin and leptin etc. in the early phase of the disease and application of suitable corrective measures through life-style management and drugs may prove more beneficial instead of searching for a ‘magic bullet’ to cure AD. This will require a concerted and multi-centric effort by neurologists, geriatricians, psychiatrists and basic neurobiologists to change the existing dogma of AD pathogenesis and treatment.

A complex interplay of issues must be addressed from the need for equitable medical systems for the smaller young healthy population [6] and effective treatments and limitations of current therapies [7, 8] to the ethics of anti-ageing treatments [9, 10], and so on. The complexities of such issues highlight the need for interdisciplinary collaborations to create novel solutions, which would otherwise be impossible for single disciplines. Even though interdisciplinary research has been growing [11], there are barriers such as lack of funding [12], lack of institutional support [13], and the associated lower productivity due to fewer papers published [14], which can all hinder the development of integrative interdisciplinary research. In this paper, we highlight some of the limitations and shortcomings faced in Alzheimer’s disease research and treatments, and then suggest that interdisciplinary research can overcome these issues.

## The need for interdisciplinary research in understanding AD

One of the major hurdles in finding an effective treatment for AD is that we still do not have a comprehensive definition of AD. The combination of pathological and clinical features, however, does not overcome the issue of defining the disease—defining a “disease” requires identification of the causes [15]. It has long been thought that AD is caused by the deposition of amyloid-β [3, 4]. However, amyloid-β can be found in healthy older subjects [16, 17], and there seems to be little to no correlation between amyloid load and the degree of cognitive decline in AD patients [18, 19]. Other markers like tau and neurofibrillary tangles correlate better to cognitive decline in AD patients, yet this still does not establish a causative role, and may simply reflect a pathological feature of another causative process [15]. This lack of understanding of the causes of AD brings difficulties in itself, yet it is further compounded by the fact that it prevents the development of good experimental models [3, 4]. Transgenic mice are one of the most widely used models in AD, and their use has been extensively discussed by Drummond and Wisniewski [20], yet they and others have noted issues in the lack of consistency of these experimental models or their lack of relevance (e.g., dependence on familial AD mutations but most cases tend to be sporadic). However, more aetiologically relevant models like natural ageing models [21] face issues of time and cost, and while they do show impaired memory reminiscent of AD [22], they do not necessarily spontaneously develop AD-like pathologies [23]. Overall, the lack of understanding of AD and the lack of good experimental models has severely hindered progress in AD research.

AD is complex with multifactorial pathologies involving the interplay of thousands of factors and interactions [24, 25]. Therefore, those studying AD can easily get lost in the magnitude of information, leading to intuition-based research rather than disciplinary-based [26], which in turn leads to the issues mentioned above. The increased availability of omics tools [27] and computational power have provided researchers with tools to approach these problems in a more disciplined manner, yet the correlative nature of such experiments might lead to some scepticism [28]. On the other hand, computational analysts have yet to effectively harness the wealth of data generated by basic and clinical researchers [26]. This highlights the need for interdisciplinary collaborations involving computational analyst and modellers together with basic and clinical researchers to make sense of the data, allowing the generation of new hypotheses and for better computational and experimental models of AD. Harnessing the power of sophisticated computational techniques together with contextualization of both the input and output data could be the key to successfully addressing some of the most difficult problems facing AD research. As an example of the power of collaboration, multidisciplinary researchers from physics, neuroinformatics, and physiology have developed an in-silico model of hippocampal neurons that respond identically to biological neurons under a wide range of stimulations, which has implications in both modelling of AD and for finding treatments [29]. Overall, there is a good case for the use of a combination of tools from multiple disciplines to develop models of AD and to unravel the complexities of its pathology.

## The need for interdisciplinary research to address cost and equity in the treatment of AD

One of the problems that has emerged from our lack of understanding of the causes of AD is that we do not have good experimental models, leading to high failure rates of AD drugs in clinical trials (99.6% failure rate) [7]. This lack of understanding also limits our ability to find biomarkers that could indicate if a drug candidate is ineffective before reaching expensive Phase III clinical trials [30]. Overall, these and other issues have made the development of AD drugs incredibly expensive, which vastly exceeds the cost of developing drugs in other therapeutic areas [31]. Consequently, this results in high costs to patients and healthcare systems [32, 33], making it one of the most expensive diseases even when compared to heart disease or cancer (https://act.alz.org/site/DocServer/2012_Costs_Fact_Sheet_version_2.pdf?docID=7161). This problem is compounded by the equity of AD treatments between the rich and the poor, how do healthcare systems deal with cost of treating those who cannot afford to pay, and what does it mean for society when only the rich can afford treatments? Furthermore, an aging population brings with it a smaller support ratio of younger healthy population (paying tax and/or insurance) to older population suffering from AD, which in turns further threatens the sustainability of a healthcare system [34].

Issues like the above require interdisciplinary teams to address. Economists would be needed to understand the economics of very expensive treatments, as well as the implications of how government bodies, institutes, and private pharmaceutical companies invest in AD treatments. Economists working with scientists and policymakers can then develop strategies to better manage the costs. Operational researchers working with scientists, clinicians, and administrators can further develop optimal workflows that could eventually reduce the cost of treatments. Sociologists and communicators can also play crucial roles in addressing and overcoming the difficulties in implementing such strategies. For example, cholinesterase inhibitors (CIs) and the NMDA receptor antagonist memantine can be used in the early stages of AD and are usually well-tolerated, yet despite multiple pharmacoeconomic studies, uncertainty in detecting the improvements has meant physicians are reluctant to prescribe them or insurers are not willing to cover the costs [9]. Based on good scientific reasoning and economic analysis, strategies could be implemented and properly communicated to drive policies and address this disconnect. Overall, the issue of cost and equity of AD treatments can only be effectively addressed if multidisciplinary teams are set up to approach them in a holistic manner.

## The need for an interdisciplinary research approach for AD treatment and care

For an effective implementation strategy, we need to properly consider both treatment and care of AD patients. We already covered this briefly in the previous discussion on understanding the pharmacoeconomics of AD treatments and development. Beyond economics, we also need to understand human behaviour, beliefs, and perceptions (among others). For example, Deep Brain Stimulation (DBS) has been gaining attention as a potential therapy for AD and dementia [21, 35-37] but understanding the reluctance of patients to undergo DBS surgery also needs to be considered. It has been found to be one of the most effective treatments for Parkinson’s disease [38], yet a huge proportion of patients with advanced Parkinson’s disease are reluctant to undergo DBS due to the fear of complications and costs [39]—these problems would also be present if DBS is approved as a clinical treatment for AD. Beyond the treatment of the patient, we also need to consider their family and caregivers, as they are crucial for a successful implementation. In the above example, encouragement from the family would be a big contributing factor to overcome the reluctance for DBS [39]. Similarly, addressing the caregiver burden has been shown to be important for DBS patients [40]. Furthermore, given that AD affects the patient’s mental functioning, preference of advance directives is crucial for respecting the patients autonomy [41]. Understanding both family and caregiver perceptions and concerns about the treatments is, therefore, an integral part of AD treatments.

On the one hand, these issues do not belong in the traditional fields of STEM (Science, Technology, Engineering, Mathematics), but do benefit from the input of STEM research. The tremendous development of electronics, computer science, digital technology and robotics have opened up new vistas in the assessment and monitoring of cognitive impairment and providing assistance to the subjects of dementia to improve the quality of life. Thus, computer-based and various app-based assessment methods of cognitive impairment, and interactive computerized brain games and cognitive training tools may substantially assist the clinicians and caregivers to monitor the progress of the dementia and in addition may alleviate the memory impairment, behavioural alterations and emotional distress of such patients with improvement of functional ability [42-44]. On the other hands, these issues require the know-how of disciplines in HASS (Humanities, Arts, Social Sciences), and in turn, the outcomes help to steer the direction of STEM research. However, understanding the needs of patients and caregivers is only the first step to developing the solutions. For example, potential difficulties in the uptake of AD treatments and the difficulties faced by caregivers could direct research into areas that lessen the resistance from both patients and caregivers (e.g., non-invasive DBS or prioritizing the development of treatments that do not affect impulse control). Similarly, difficulties faced by caregivers could be eased through advancements in technology. Overall, there is a need for different disciplines to work together, not only to develop treatments for AD, but also to develop better approaches to treatments and to better support caregivers.

## The need for international organizations to advance interdisciplinary research

In the previous sections, we argued that integrative interdisciplinary research is crucial for the successful discovery and implementation of AD treatments, yet barriers such as lower funding [12], different cultures across disciplines [45], lack of institutional support [13], and fewer published papers [14] present huge hurdles for researchers from different disciplines working together. To address these issues, we argue that there needs to be a push from international organizations towards promoting interdisciplinary research. One example is the eLife ambassador program, which is working to create both online and offline platforms to support scientists from different disciplines and from different cultures and backgrounds to develop “ways to accelerate the transition to more open, collaborative, and reproducible science” (https://elifesciences.org/inside-elife/2a8f1672/elife-ambassadors-an-invitation-to-take-part-in-2019). Within the eLife ambassador program, the Collaborative Science Initiative further aims to promote interdisciplinary research by developing tools and training materials based on the feedback from scientists on their concerns about collaborating to overcome some of the barriers in collaborative research. Publishers and journals could work towards publishing articles addressing these issues. For example, PLOS Computational Biology has published multiple “Ten Simple Rules” articles on collaborative research including cross-disciplinary collaborations [46], multi-site collaborations [47], and collaborations in general [48]. Although these papers provide crucial information for scientists to establish good practices in collaborative work, more can be done to promote cross-disciplinary research. A call could be made for interdisciplinary research papers working towards removing the stigma of lower productivity or highlighting the need for cross-disciplinary research, which in turn would play a huge role in developing better collaborations between disciplines. Regardless, many of the difficulties of interdisciplinary research remain, which inevitably takes time and resources to tackle. The lower funding and lack of support from institutions make interdisciplinary work incredibly difficult. Policies that incentivize collaborative work, especially between STEM and HASS disciplines would go a long way to foster interdisciplinary teamwork [49]. International societies can play a role by providing funding specifically allocated to interdisciplinary research, as well as lobbying funders to promote cross-disciplinary work. Special issues in the journals of these societies could also incentivize interdisciplinary research. Overall, professional societies need to take an active role in supporting cross-disciplinary collaborations.

Societies such as the International Society on Aging and Disease (ISOAD) (www.isoad.org) and UNESCO Executive Committee on Anti-aging and Disease Prevention (www.aginganddisease.org/EN/10.14336/AD.2019.1230) are primed to push for increased interdisciplinary collaborative research, with many of the above suggestions fitting within the goals of ISOAD. First, developing platforms and infrastructure for collaborative research would strengthen the goal of “facilitat(ing) interactions among investigators in ageing and disease”. Conferences that gather researchers from different fields to meet other researchers and share their work is a good first step to developing interdisciplinary collaborations. However, beyond that, more can be done to develop online platforms to encourage interactions and stimulate discussions. The challenge would be, of course, to develop a culture in which researchers habitually use these platforms, which will take time, effort, and willpower. Call-out for papers on interdisciplinary research or special issues focusing on interdisciplinary work would not only incentivize cross-disciplinary research that could lead to novel discoveries, but also fits into the society’s goal to “disseminate recent discoveries of the interaction between ageing and age-related disease through its journal, Aging and Disease”. Given the fact that Aging and Disease already publish papers on humanities-based topics such as ethics [50, 51], further subsections focusing on social sciences and humanities could be developed that would also expand the readership of the journal to cross-disciplinary fields. However, this might be ambitious as it requires the recruitment of editors familiar with the field and might be hard to maintain metrics due to the lower citation rates of papers from humanities and social sciences [52, 53]. Lastly, a goal of ISOAD is to “lobby funding agencies about the importance of the study of ageing and disease”. Indeed, members of the ISOAD committee have written about the critical need to increase funding and incentivize research into age-related diseases [54]. Focus can, therefore, be placed on lobbying funding bodies to provide specialized funds to incentivize interdisciplinary collaborative research on AD. However, this may be a double-edged sword, as providing specialized funds for interdisciplinary research would mean less funding for those working on mono-disciplinary research, which might affect many of the society’s members. Overall, a transition to more interdisciplinary research has its pros and cons. Nevertheless, it would take the willpower of both leaders and members of the society and the research community to subscribe to this approach. Metrics in which researchers are judged upon need to be re-evaluated and proper incentive systems need to be developed to compliment this shift.

## Conclusions

In this commentary, we have argued that interdisciplinary collaborative research is needed in order to address some of the most difficult yet pressing issues in AD research. We have suggested that international societies, together with journals and publishers, have a huge role to play in achieving a landscape in which interdisciplinary research can thrive. We have highlighted some of the difficulties and hurdles that stand in the way of achieving these goals and have suggested that this transition needs willpower and leadership in the research community together with changes in the current metrics and systems of incentivization. Although these goals are difficult and ambitious, we believe that these approaches can help tackle complex research issues. We are encouraged by the current trend towards greater interdisciplinary research, and optimistic that more collaborative work will emerge to address some of the most important issues facing AD research.
